# Notes and Notices

**Published:** 1888-02

**Authors:** 


### NOTES AND NOTICES.

THE CHOICE OF A DOCTOR.—Hahnemann wrote: "Search for a man of sound common sense, who takes great pains to ascertain the truth of all he hears and says. * * * Before you finally fix on him, see how he behaves to the poor, and if he occupies himself at home, when unseen, with some useful work!"

